# Novel insights into pore-scale dynamics of wettability alteration during low salinity waterflooding

**DOI:** 10.1038/s41598-019-45434-2

**Published:** 2019-06-25

**Authors:** Rimsha Aziz, Vahid Joekar-Niasar, Pedro J. Martínez-Ferrer, Omar E. Godinez-Brizuela, Constantinos Theodoropoulos, Hassan Mahani

**Affiliations:** 1 University of Manchester, School of Chemical Engineering and Analytical science, Sackville St, Manchester, M139PL United Kingdom; 2 Manchester Metropolitan University, Centre for Mathematical Modelling and Flow Analysis, Chester Street, Manchester, M1 5GD United Kingdom; 3 Shell Global Solutions International B.V., Grasweg 31, 1031 HW Amsterdam, Netherlands

**Keywords:** Crude oil, Chemical engineering

## Abstract

Low salinity waterflooding has proven to accelerate oil production at core and field scales. Wettability alteration from a more oil-wetting to a more water-wetting condition has been established as one of the most notable effects of low salinity waterflooding. To induce the wettability alteration, low salinity water should be transported to come in contact with the oil-water interfaces. Transport under two-phase flow conditions can be highly influenced by fluids topology that creates connected pathways as well as dead-end regions. It is known that under two-phase flow conditions, the pore space filled by a fluid can be split into flowing (connected pathways) and stagnant (deadend) regions due to fluids topology. Transport in flowing regions is advection controlled and transport in stagnant regions is predominantly diffusion controlled. To understand the full picture of wettability alteration of a rock by injection of low salinity water, it is important to know i) how the injected low salinity water displaces and mixes with the high salinity water, ii) how continuous wettability alteration impacts the redistribution of two immiscible fluids and (ii) role of hydrodynamic transport and mixing between the low salinity water and the formation brine (high salinity water) in wettability alteration. To address these two issues, computational fluid dynamic simulations of coupled dynamic two-phase flow, hydrodynamic transport and wettability alteration in a 2D domain were carried out using the volume of fluid method. The numerical simulations show that when low salinity water was injected, the formation brine (high salinity water) was swept out from the flowing regions by advection. However, the formation brine residing in stagnant regions was diffused very slowly to the low salinity water. The presence of formation brine in stagnant regions created heterogeneous wettability conditions at the pore scale, which led to remarkable two-phase flow dynamics and internal redistribution of oil, which is referred to as the "pull-push" behaviour and has not been addressed before in the literature. Our simulation results imply that the presence of stagnant regions in the tertiary oil recovery impedes the potential of wettability alteration for additional oil recovery. Hence, it would be favorable to inject low salinity water from the beginning of waterflooding to avoid stagnant saturation. We also observed that oil ganglia size was reduced under tertiary mode of low salinity waterflooding compared to the high salinity waterflooding.

## Introduction

### Pore-Scale Mechanisms of Low Salinity Waterflooding

Low salinity waterflooding is a relatively new enhanced oil recovery (EOR) technology in which the ionic strength and composition of injection water are designed to achieve an additional oil recovery. Low salinity waterflooding has been a point of discussion since 1967^1^. The potential of this technology was first demonstrated by Tang and Morrow^2^ through experiments, where up to 15% additional oil was produced from the core with substantial reduction of salinity of the injecting water^2^. In sandstone reservoirs, the injection water should have a much lower salinity compared to the formation brine, while in carbonate reservoirs that cannot be necessarily the case due to the fundamental differences in geochemistry and rock-fluid interactions. Several factors such as rock heterogeneity, mineralogy of rock, brine composition and crude oil chemistry control performance of low salinity waterflooding^3–5^. The general consensus in literature supports that low salinity waterflooding changes wettability from oil-wet to more water-wet state^2,6–8^ which leads to additional oil recovery as shown in some experimental studies at field scale, core scale and pore-scale^2,9–12^.

Wettability is a key property of the solid-fluid system, which controls the fluid configuration in the pore space. Therefore, alteration of wettability will cause a substantial change in fluid occupancy and its behaviour^3,12,13^. The wettability state of a reservoir is a result of a complex interaction between crude oil, brine and rock (COBR)^3,7,10,14^, therefore, many mechanisms have been proposed to explain this phenomenon^5,8^. The mechanisms can be divided into two categories: liquid-liquid and solid-liquid interactions^8^. Liquid-liquid mechanisms include a) osmosis effect and b) visco-elastic interface. In the osmosis effect the oil acts as a semi-permeable membrane and oil droplets are relocated under osmosis gradient, absorbing water to create new pathways for oil recovery^5^ and in the visco-elastic behaviour of interface, the rigidness of the interface decreases due to the low salinity water^8^. Solid-liquid mechanisms include a) double layer expansion and b) multi-ion exchange. Under low salinity conditions, the double layer expands due to increased repulsive electro-static forces leading to the increase of film thickness^7,10,15^ and reduces the interaction between crude oil and rock;^7,15–17^ ultimately changing wettability towards more water-wet conditions^2,6,7,10,15,18^. In multi-ion exchange the divalent ions are desorbed under low salinity water and are replaced with monovalent ones^19^. Divalent ions bond to the surface of the clay and to the polar compounds found in the crude oil, leading to an oil-wet state^19^. Therefore, the removal of these ions leads to a more water-wet state, as the adhesion between the rock and the crude oil decreases^10,20,21^. Note that the multi-ion exchange, double layer expansion, pH variation and contact angle change due to the low salinity waterflooding can be all theoretically explained in a single system of theories as discussed in the literature^15^. Other possible mechanisms postulated in the literature can be found in the recent review papers^5,8^.

Some experimental studies show that there is a shift in relative permeability and capillary pressure (continuum scale parameters) as a result of wettability alteration (sub-pore scale process) under low salinity waterflooding^22–24^. However, the impacts of wettability on pore-scale two-phase flow processes responsible for the observed trends at continuum scale are not well understood^8^. This gap in the physical scale urges the necessity to develop pore-scale models to investigate the role of transport of low salinity water and its mixing with the formation brine as well as multiphase flow dynamics induced by wettability alteration.

The dynamics of two-phase flow under wettability alteration induced by the low salinity waterflooding is still unknown to a large extent and very few studies have provided some insights. Low salinity waterflooding can be injected into porous medium from the beginning (secondary mode) or after high salinity waterflooding (tertiary mode)^25–27^. In experimental studies, secondary mode of low salinity waterflooding has produced a higher oil recovery than tertiary mode^2,16,25–29^. Even though secondary mode of low salinity waterflooding has always produced significant additional oil recovery compared to high salinity waterflooding, tertiary mode of low salinity waterflooding has not always resulted in the same way^17,30,31^. This is due to the state of oil present at pore-scale during secondary and tertiary mode. Under secondary mode of low salinity waterflooding, oil is found as a continuous phase, since low salinity waterflooding improves sweep efficiency, this leads to higher oil recovery^30,32^. Under tertiary mode of low salinity, oil is found as discrete oil clusters (ganglia) and cannot be remobilised easily^30^. Favourable oil recovery under tertiary mode of low salinity waterflooding has been recorded when oil banking has been observed^12,33^. Overall, low salinity waterflooding studies have shown a significant reduction of oil ganglia size in experimental pore-scale studies^11,12,34–37^. An experimental study on a 2D micro model have shown that high salinity waterflooding displaces oil from larger pores which allows the smaller pores to be more accessible for wettability modification or osmosis gradients (salt gradient)^38^. This is further confirmed by other experimental studies in which low salinity waterflooding has displaced a large fraction of the smallest pores in the porous medium^33,39^. Since wettability alteration in experimental studies cannot be controlled or systematically changed and is highly dependent on COBR interaction; hence experimental pore-scale studies have not been able to evaluate the optimal wettability conditions for low salinity waterflooding under tertiary mode. In a recent pore network modelling study, in which pre-defined wettability alteration mechanisms were simulated, it was concluded that a greater change in wettability towards water-wet conditions leads to higher oil recovery in the tertiary mode^40^. This is interesting because without wettability alteration, the optimum wettability is neutral wet to weakly-wet^3^, but in case of wettability alteration, stronger water-wet conditions are more preferred^40^. This discrepancy in conclusions depending on the flooding conditions has not been clearly addressed in the literature and pore-scale simulations can potentially explain them.

### Transport of low salinity water through the porous media and heterogeneous wettability alteration

After high salinity waterflooding, the pore space is filled with oil and high salinity water which may have reached the steady-state flow. The pore space occupied by high salinity water can be decomposed to flowing regions (which contribute to flow) and stagnant regions (which are hydro dynamically inactive) as they are mostly situated in the dead-end regions of the water phase^41–44^. These two regions have different transport time scales. Thus, after injection of low salinity water, the high salinity water residing in the flowing regions are pushed away by the injected low salinity water, while the high salinity water in the stagnant regions remains for very long time and mix with low salinity water by counter-diffusion. In theory, low salinity water can reach flowing pathways within one pore volume and will take many more pore volumes to mix low salinity water into high salinity water in the stagnant region^44^. Therefore, stagnant regions will experience wettability changes much later in time than flowing pathways, thus creating heterogeneous wettability conditions at the pore-scale. Two-phase flow dynamics through heterogeneous/mixed wettability porous medium is unknown in literature and very few studies have provided some theoretical understanding of flow dynamics under such conditions^45^. Modelling of low salinity waterflooding has been attempted at continuum scale^22,46–49^, these studies have assumed stagnant regions have no significant influence on the flow dynamics.

### Objectives

Mixing of low salinity water with the resident high-salinity water directly influences the local wettability alteration. Due to the heterogeneous spatial distribution of pore-scale velocities, transport and mixing would not occur homogeneously over the void space and it is important to capture the impact of transport of low salinity water on wettability alteration under two-phase flow conditions. Therefore, it is important to develop a computational fluid dynamic model to capture two-phase flow dynamics, transport and mixing of low salinity with high salinity water as well as wettability alteration. Also, we aim to address the signature of stagnant regions on wettability alteration and oil recovery under secondary and tertiary modes.

Our final objective is to delineate the role of stagnant regions and wettability alteration on two-phase flow dynamics and fluids redistribution.

## Results and Discussion

### Additional oil recovery by low salinity waterflooding

Simulations of low salinity waterflooding were performed for the three different initial conditions, explained in the previous section. Under high-salinity conditions the contact angle was 140° and under low salinity conditions contact angles of 30° and 60° were assumed.

**Figure 1:**
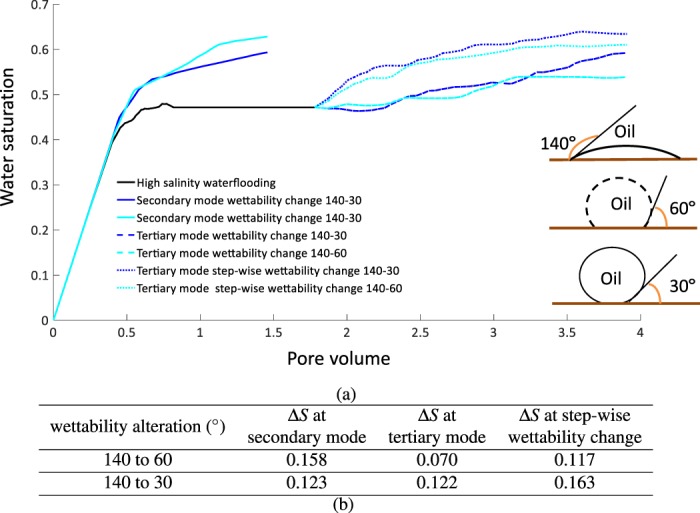
(**a**) Water saturation (y-axis) against pore volume (x-axis) for high salinity waterflooding, secondary mode of low salinity injection and tertiary modes of low salinity injection and tertiary mode with step-wise wettability change for contact angle 30 and 60. (**b**) Additional oil recovery for three cases of initial condition and two wettability alteration scenarios, additional oil recovery was calculated by taking the difference between the final water saturation at the end of high salinity waterflooding and the respective cases.

Under high salinity waterflooding 0.47 of oil saturation was recovered. Then the additional oil recovered by injection of low salinity water in secondary and tertiary modes were estimated as shown in Fig. 1a,b. Figure 1 shows the change of water saturation with pore volume. Secondary mode under both wettability alteration scenarios have shown a better oil recovery compared to the tertiary mode. In the secondary mode, an additional oil recovery equivalent to the 12.3% and 15.8% of pore volume was produced for both wettability scenarios. Tertiary mode produces between 7% and 12% pore volume of additional recovery, see Fig. 1b. Interestingly the contact angle of 30° seems to be more favorable for the tertiary mode, while the contact angle of 60° is more favorable for the secondary mode.

Figure 1b shows the ultimate additional oil recovery for different cases. The tertiary mode simulations were run until the steady-state saturation was reached. The additional oil recovery under low salinity waterflooding in both modes is due to the improved sweep efficiency, caused by the wettability change, as more pores are invaded^16,25,30,45,50^. Not all studies in the literature have shown favourable oil recovery despite the low salinity water injection and wettability alteration under tertiary mode^5,10,12,33^.

These results are in line with many experimental studies on low salinity waterflooding, where injecting low salinity in the secondary mode is more favourable for oil recovery than tertiary mode^16,17,25–27,30,31,51,52^. However, Fig. 1b clearly shows under tertiary mode when the stagnant regions have been discarded by abrupt change of contact angle from oil-wet to water-wet, the additional oil recovery has been significantly increased, which can potentially highlight the undermining role of stagnant regions.

### “Pull and Push” Mechanism and Fluids Redistribution

**Figure 2:**
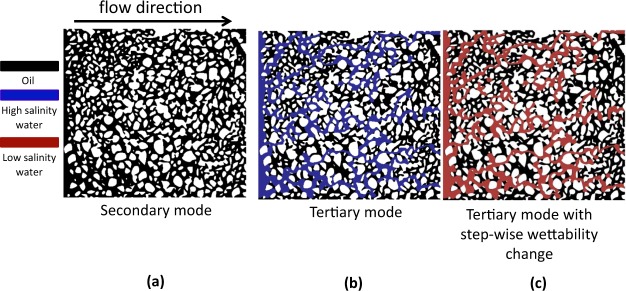
(**a**) Initial conditions for the low salinity waterflooding in the secondary mode, (**b**) Initial condition for the low salinity waterflooding in the tertiary mode, (**c**) Initial condition for the low salinity waterflooding with step-change of wettability.

The initial conditions for secondary and tertiary low salinity waterflooding have been shown in Fig. 2a–c. Under secondary mode, the oil is found in the porous medium as a continuous phase; however, in tertiary mode the porous medium is filled with oil ganglia and high-salinity water, see Fig. 2b. Since wettability alteration targets change of capillary forces and can potentially redistribute the fluids, under tertiary mode the oil phase has more flexibility to redistribute inside the porous medium that can make additional oil recovery more difficult to happen. Another aspect of low salinity waterflooding in the tertiary mode is the heterogeneous distribution of salinity which can lead to mixed wettability in the water-filled regions. This has not been addressed in the literature.

In the tertiary mode, low salinity water fills the pore space initially filled by the high salinity water and consequently the wettability changes along the transport of the low salinity water. Previous studies of transport under two-phase flow conditions illustrate the transport within the water-filled area does not happen homogeneously and some regions are more advection controlled and some other regions such as dead-end regions are predominantly diffusion controlled^41–44^.

**Figure 3:**
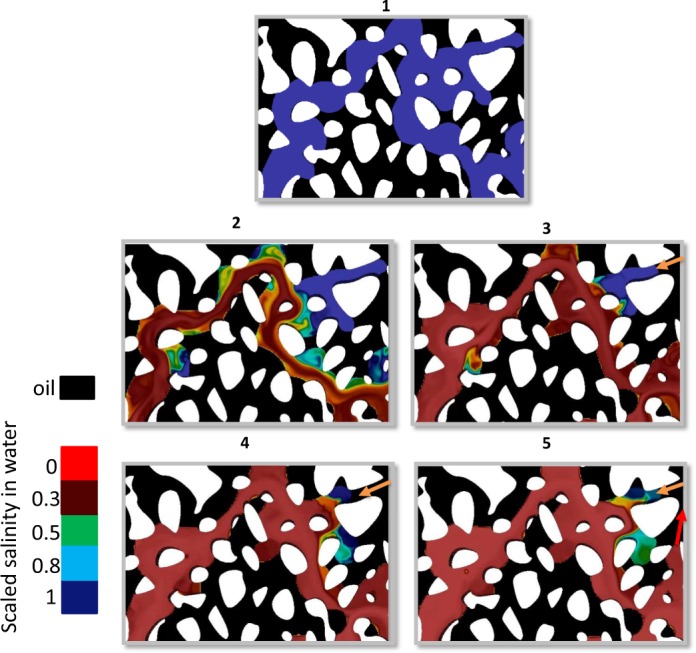
A snapshot serie of oil and water redistribution during the tertiary mode of low salinity waterflooding (time sequence is shown at the top of each snapshot). Oil is shown in black colour and high salinity water is shown in (dark) blue colour. Other colors correspond to different salinity values in water. The stagnant regions remain filled with the high salinity water (oil-wet), while the main flow path has been filled by the low salinity water (water-wet). As a result of mixed wettability destabilisation of capillary interfaces, fluids redistribution takes place. Water *pulls* out from the high salinity water-filled regions (in direction of yellow arrow in frame 2) and water *pushes* the oil out from nearby pores (in direction of the red arrow in frame (5).

The dead-end regions have been shown in dark blue color in Fig. 3, frames 2 to 5. Under low salinity waterflooding in the tertiary mode, pores which are part of flowing pathways (red regions) will experience wettability changes at an earlier time compared to the dead-end regions, which leads to different local wettability conditions with parts of the water topology remaining oil-wet (dead-end regions) and other regions experience water-wettability (flowing regions). Note that in reality the spatial variation of wettability conditions will depend on a) size of dead-end clusters which can highly vary depending on the variation in pore morphology of the rock, b) the difference between the wettability alteration time scale (which has not been considered in this study) compared to the diffusion time scale in dead-end regions which can be potentially much larger than the dead-end regions in the presented simulations.

Induced wettability alteration at the capillary interfaces, destabilises the saturation profile established under high-salinity water flooding. As a result of the wettability alteration the capillary pressures at the main menisci (interfaces between oil and water) which change towards more water-wet, will assist water invasion to new regions during waterflooding. Since stagnant regions are oil-wet, water would *pull* out from the dead-end regions (arrows in Fig. 3, frames 3 and 4) and in some new regions water would *push* the oil out of pores (the red arrow in Fig. 3, frame 5).

Note that this fluids’ redistribution is possible due to the spatial distribution of positive and negative capillary pressures. Under oil-wet conditions, the capillary pressure at menisci (difference between the oil and water pressures) is negative and in order for water to invade a new pore, extra energy is required to overcome the entry capillary pressure of a pore. Under water-wet conditions the difference between the oil and water pressures becomes less negative or even positive, which facilitates water filling (imbibition) events into new pores. Based on the simulation results, we hypothesize that stagnant regions enhance internal counter-current flow and redistribution of fluids, which undermine the performance of low salinity waterflooding. In our simulations, this *Pull-Push* dynamics was only unique to low salinity injection in tertiary mode due to the presence of stagnant saturation and was not observed in the secondary mode.

In order to confirm whether the stagnant zones limit the reach of low salinity waterflooding or not, a hypothetical case was created with the fluids distribution identical to the start of tertiary low salinity waterflooding. However, we initiated the simulation by setting all the water-filled regions to low-salinity water concentration and its corresponding contact angle. Therefore, wettability changed all around the water topology as a step-function from the oil-wet to the water-wet, instantaneously, see Fig. 2c, Thus, the stagnant regions were fully eliminated. The simulations were run until the saturation reached the steady state. The additional oil recovery are shown in Fig. 1a,b. Both scenarios of wettability change (60° and 30°) showed a higher oil recovery compared to the tertiary mode of low salinity waterflooding with presence of stagnant saturation.

This clearly indicates that stagnant regions under high salinity waterflooding can decrease the potential of low salinity waterflooding and delay the ultimate oil recovery. Note that here this negative impact of high-salinity regions is not only due to the mixing, but due to the induced fluids redistribution as a result of local wettability alteration. These results provide additional potential explanations to why the secondary mode of low salinity waterflooding is more favourable for oil recovery. From these results, it can be concluded that it is much more efficient to inject low salinity water from the beginning, this will not only eliminate the drawback of “stagnant regions” but due to the better connectivity of oil at the secondary mode, can potentially lead to higher oil recovery. The presented results are focused on pore-scale fluids distributions. However, the negative impact of stagnant regions can be found at larger physical scales, where the rock heterogeneity may be found. The formation heterogeneity would cause high-salinity water to remain in low permeable regions and gradual mixing with the low salinity water in the main flow path. This would deplete the quality of low salinity water and impede the wettability alteration.

These results strongly recommends to include the stagnant regions in larger scale simulations of low salinity waterflooding, which is missing in previous continuum-scale modelling studies^22,46–49^. Former experimental and modelling studies suggest that the stagnant saturation is a function of the total injecting phase saturation^42–44^. Therefore, the response to low salinity waterflooding can vary depending on the starting total water saturation of high salinity water. The highest stagnant saturation was observed in intermediate water saturation, which is the injection point for tertiary mode of enhanced oil recovery method^53^.

Please note that at the larger physical scale, in case of larger dead-end regions and higher salinity contrasts, the mixing of high-salinity water with the low-salinity water, increases the net concentration of the flowing water. Hence the performance of the low salinity water is impeded by the deterioration of the quality of low salinity water, which requires a larger scale simulation at the continuum scale.

### Spatial Distribution of Filling Events during Tertiary Low Salinity Waterflooding

**Figure 4:**
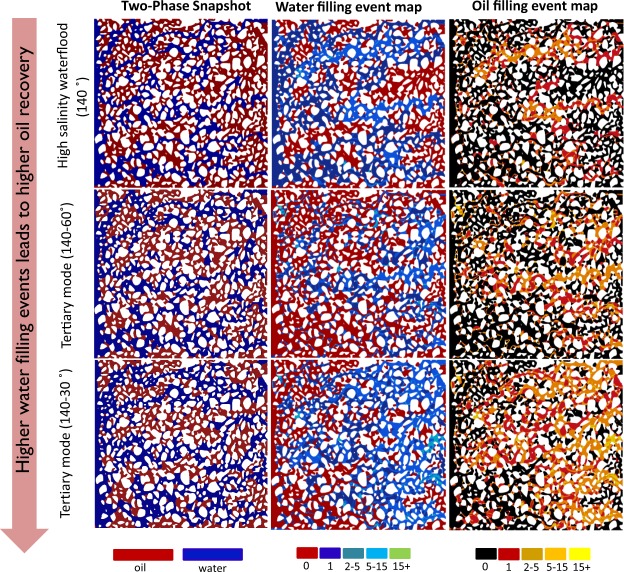
Pore-filling events maps: left column shows final oil (red) and water (blue) distribution at the end of high salinity waterflooding (top row), tertiary mode low salinity waterflooding for ultimate contact angles of 60° (middle row) and 30° (bottom row). In the same sequence of simulations, the middle column shows the maps of water filling events. The right column shows the map of oil filling events. Lighter colors correspond to higher number of events for a given position.

As explained in the methodology section, we analysed the spatial distribution of the pore-filling events by comparing each two consecutive simulation time steps. These event maps provide insights into the fluids movements and their spatial distribution during high-salinity and low-salinity water flooding as shown in Fig. 4.

Water filling events are shown in the middle column of Fig. 4 and oil filling events are shown in the right column. These maps clearly highlight how much more active the porous medium is under low salinity waterflooding in comparison to high salinity waterflooding. During the same amount of injected volume, the porous medium experienced up to 15 oil/water pore filling events under the low salinity waterflooding in comparison to the high salinity waterflooding, see the middle and right columns of Fig. 4. Tertiary mode (140–30°) experienced a higher number of water/oil filling events compared to the tertiary mode (140–60°) and it produced twice as much oil (see Fig. 1b). The number of oil filling events and water filling events in the porous medium were summed up and the difference between them was calculated to estimate the net number of events for a given simulation. Under tertiary mode 140–60° the water filling events were less frequent compared to the tertiary mode 140–30°. This was due to the larger capillary force induced under 140–30° which led to more displaced oil. Hence, we can conclude that a higher number of water filling events would lead to a greater oil recovery. Figure 4 clearly shows the large amount of oil filling events that indicate a significant spatial redistribution of oil in the porous medium due to wettability alteration experienced under low salinity waterflooding.

**Figure 5:**
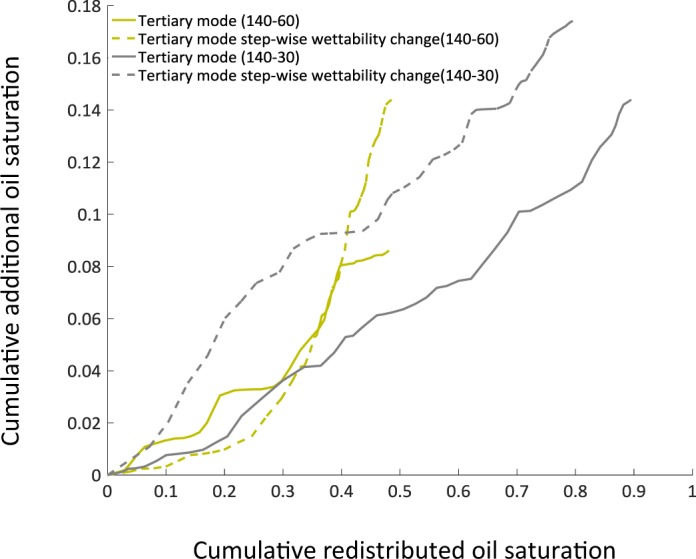
Cumulative of additional oil saturation produced from the start of low salinity water injection till the end for tertiary mode for contact angles of 140–30/60° (solid lines) and step-wise wettability change of 140–30/60° (dashed lines) is on y-axis. Cumulative of redistributed oil saturation from start till the end of low salinity injection is shown for the same cases. Indicative of high degree of oil redistribution in porous medium under cases with stagnant regions (tertiary mode simulations).

Oil recovery is mainly driven by water filling events which indicate higher mobilisation efficiency of oil at pore scale. Under wettability alteration, due to “pull-push” behaviour of water, oil can migrate to larger pores by displacing high salinity water. This will cause spatial redistribution of oil. To show the impact of pull-push behaviour on redistribution of fluids, the cumulative additional oil saturation was plotted against the cumulative redistributed oil saturation for both wettability scenarios in the tertiary mode as well as the step-wise wettability alteration model in Fig. 5. The cumulative redistributed oil, presented in terms of saturation, is the amount of oil redistributed internally in the porous medium from the time of starting low salinity water flooding to the end of low salinity water flooding. Since the oil can be moved internally this does not mean that all the oil would be produced from the outlet boundary. Looking at Fig. 5, to achieve the same additional oil recovery in different cases, the tertiary mode simulations have gone through larger redistribution of fluids that indicates the lower performance of the dynamic system compared to the cases where stagnant regions do not exist. For the case of 140–60°, initially the step-wise wettability alteration case shows a suppressed production however, at the later stage, the oil production overtakes the tertiary mode case (Fig. 5).

### Oil Ganglia Size after Low Salinity Waterflooding

**Figure 6:**
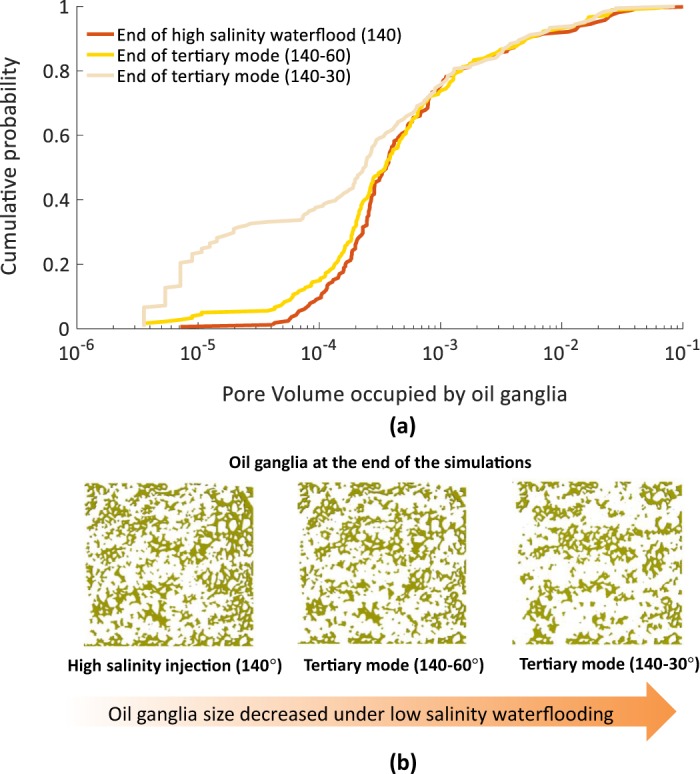
(**a**) Oil ganglia size on x-axis shown in terms of the amount of pore volume occupied by oil cluster with cumulative probability on y-axis at the end of waterflooding in high salinity waterflooding and tertiary mode of low salinity. (**b**) Snapshot of oil ganglia present at the end of the simulations.

Figure 6a shows the cumulative probability of the size of oil ganglia at the end of high salinity waterflooding and low salinity waterflooding in the tertiary mode. Figure 6b shows the snapshots of the oil ganglia at the end of the simulation. After low salinity waterflooding the size of oil ganglia decreases. This is clearly shown when the probability of small ganglia size increased under wettability alteration scenario of 140–30°. This trend has also been seen in recent pore-sale studies of low salinity waterflooding^11,34–36^. Our results show that greater wettability alteration (140–30°) leads to more breakdown of oil ganglia compared to the case of 140–60°. This is due to the higher capillary forces induced in 140–30° which remobilises more oil and leads to a higher recovery factor. Oil banking is absent in our simulations due to a high viscosity ratio, which has been similarly reported in the experimental results^54^.

**Figure 7:**
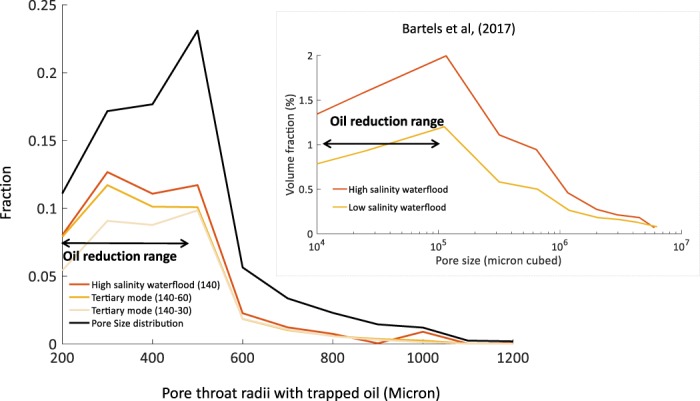
Pore size distribution of trapped oil at the end of simulations.

We also investigated the displacement of oil under low salinity water by calculating the pore size distribution where the oil is trapped after high salinity and low salinity waterflooding, refer to Fig. 7. Figure 7 shows greater fraction of the smaller pores have been invaded under low salinity waterflooding as wettability is altered towards more water-wet state. These results are well supported by the recent experimental study^33^, where low salinity waterflooding in tertiary mode displaced oil from the narrowest pores in the porous medium. This behaviour can be explained as water becomes the wetting phase due to wettability alteration, water is imbibed into the smaller pores easier due to the support of capillary forces.

## Conclusions

We simulated coupled pore-scale two-phase flow, transport and wettability alteration using the computational fluid dynamics implemented by the volume of fluid in OpenFOAM. The objectives of this study were to show a) the importance of transport and stagnant regions in low salinity waterflooding, b) the possible redistribution of fluids during tertiary mode of low salinity waterflooding and their impact on the oil recovery, c) the impact of initial condition (secondary versus tertiary modes) on final performance of oil recovery.

We defined three wettability alteration scenarios and two different initial conditions to simulate secondary and tertiary modes. Our study showed higher oil recovery can be achieved when low salinity waterflooding was deployed in the secondary mode compared to the tertiary mode. During the tertiary mode, the presence of high salinity water and low salinity water, induces a spatial mixed wettability which can potentially be counter-productive and leads to the redistribution of fluids. As a result, oil production rate is decelerated which can impede the net ultimate production as well.

We can conclude that the presence of stagnant regions might have two negative impacts on oil recovery:

- Low salinity waterflooding in the tertiary mode initiates with destabilization of the capillary interfaces. Due to the change of wettability at these interfaces towards more water-wet (less oil-wet) case, a unique “pull-push” behavior was observed, which can be explained as following. In the tertiary low salinity waterflooding the flowing regions carry the low salinity water while the dead-end (stagnant) regions remain filled by the high-salinity water for a long time. As a result of this heterogeneous distribution of salinity, spatially-mixed wettability is resulted. After destabilisation of capillary interfaces, water from the high-salinity regions is pulled out towards the main water flow path and low salinity water pushes oil out from new regions.
- Due to the pull-push mechanism, there is a significant redistribution of fluids in the porous medium (as shown in the activity maps in Fig. 4), which do not necessarily lead to oil production. In our simulation results, volume of the redistributed oil phase is almost 5 times larger than produced oil.
- Under tertiary low salinity waterflooding ganglia size decreases and the possibility to recover oil from smaller class pores becomes higher due to the stronger capillary forces under more water-wet conditions.

## Methods

There are a number of techniques to model pore-scale multiphase flow through porous media. Notable examples are dynamic pore-network modelling^55^, Lattice Boltzmann^56^, volume of fluid method (VoF)^44,57,58^, smoothed particle hydrodynamics, and level set method. We utilize the VoF method to solve the Navier-Stokes equations corresponding to immiscible two-phase flow, available in OpenFOAM^59^. In this study, we have modelled two-phase flow using the Volume of fluid (VOF) method, coupled with the mass and momentum equation for different wettability alteration mechanisms using OpenFOAM. Although this technique has been used before to simulate the wettability alteration^32,40^, the critical role of transport and mixing and their contribution to two-phase flow have not been investigated.

### Volume of Fluid Method

Immiscible and incompressible two-phase flow in porous media was simulated using the VOF method implemented in InterFoam^59,60^, which has been successfully used in former porous media research^57,58^.

In the two-phase VOF method, each fluid phase is represented by its volume fraction: *α*_1_ represents oil and *α*_2_ (*α*_2_ = 1 − α_1_) represents water. At the interface between two fluids, there is a transition zone between the *α* phases. Thus, to avoid this transition zone, in grid cells containing intermediate values of *α* $(0 < \alpha  < 1)$, a sharp interface at *α* = 0.5 is reconstructed.

In the two-phase VOF method, each fluid phase is represented by its volume fraction: *α*_1_ represents oil and *α*_2_ (*α*_2_ = 1−*α*_1_) represents water. At the interface between two fluids, there is a transition zone between the *α* phases. Thus, to avoid this transition zone and define a sharp interface, in grid cells containing intermediate values of *α* $(0 < \alpha  < 1)$, a sharp interface at *α* = 0.5 is reconstructed. The volume fraction *α*_1_ solved using Eq. (1).

1 $$\frac{\partial {\alpha }_{1}}{\partial t}+\nabla \cdot ({\alpha }_{1}{\bf{u}})+\nabla \cdot ({\alpha }_{1}(1-{\alpha }_{1}){{\bf{u}}}_{r})=0,$$

where **u** is referred to as the velocity field and **u**_*r*_ is referred to as the relative velocity between the two fluids **u**_*r*_ = **u**_1_ − **u**_2_.

The fluids’ properties at the interface are calculated using *α*-weighted averaging:

2 $$\rho ={\alpha }_{1}{\rho }_{1}+{\alpha }_{2}{\rho }_{2}$$

3 $$\mu ={\alpha }_{1}{\mu }_{1}+{\alpha }_{2}{\mu }_{2}$$

where, *ρ*_1_ and *μ*_1_ represent density and viscosity of oil and *ρ*_2_ and *μ*_2_ represent density and viscosity of water.

Mass and momentum of the system is computed using the following Eq. (4).

4 $$\frac{\partial \rho {\bf{u}}}{\partial t}+\nabla \cdot (\rho {\bf{u}}{\bf{u}})=-\nabla p+[\nabla \cdot (\mu (\nabla {\bf{u}}+\nabla {{\bf{u}}}^{T}))]+{{\bf{F}}}_{sa}$$

5 $$\nabla \cdot {\bf{u}}=0$$

where, the body force is represented by **F**_*sa*_, which includes the interfacial forces as well.

The body force **F**_*sa*_ in Eq. (4) is defined as

6 $${{\bf{F}}}_{sa}=\rho {\bf{g}}\cdot {{\bf{n}}}_{{\bf{z}}}+\mathop{\int }\limits_{{\rm{\Gamma }}}\sigma \kappa \delta (x-{x}_{s})\hat{{\bf{n}}}d{\rm{\Gamma }}({x}_{s}),$$

where Γ is the liquid-liquid interface, and $\delta (x-{x}_{s})$ is the Dirac delta function, *κ* is the curvature of the interface, and σ is the interfacial tension between the two fluids. We used the multi-dimensional universal limiter with explicit solution (MULES) implemented in OpenFoam ver 2.3.0. There are other alternatives such as high resolution interface capturing (HRIC) and compressive interface capturing scheme for arbitrary meshes (CICSAM). The curvature of the interface is given by $\kappa =-\nabla \cdot (\frac{\nabla {\alpha }_{1}}{|\nabla {\alpha }_{1}|})$ and the unit vector **n** is defined as $\hat{{\bf{n}}}=\frac{\nabla {\alpha }_{1}}{|\nabla {\alpha }_{1}|}$. The pressure is solved using a pressure and velocity coupling PISO loop^61^.

For the two-phase flow, the contact angle *θ* at the contact line is defined as follows:

7 $$\hat{{\bf{n}}}\cdot {\hat{{\bf{n}}}}_{{\bf{s}}}=\,\cos \,\theta ,$$

where $\hat{{\bf{n}}}$ and ${\hat{{\bf{n}}}}_{{\bf{s}}}$ are vectors normal to the interface and solid wall, respectively. Note that there are other formulation which explicitly capture the dynamic contact angle in VoF simulations. Recent studies highlight the importance of dynamic contact angle to match the two-phase flow dynamics, however, in this study to discriminate the impact of flow hydrodynamics and salinity on contact angle, we assumed constant contact angle at equilibrium conditions.

Time step (*δt*) is computed using Currant number $Co=\frac{\delta tU}{\delta x} < 0.5$. *U* velocity magnitude, *δx* is the cell size. Average time step during the simulation was 10^−6^ s.

To incorporate wettability alteration in the model, a functionality between contact angle and salinity (concentration) should be predefined and inserted in the model. To our current understanding low salinity waterflooding alters wettability from oil-wet to water wet conditions, however, the final wettability contact angle is highly variable between 30−60° degree in experimental studies depending on the COBR system^2,7,17,20,21,39,62^.

**Figure 8:**
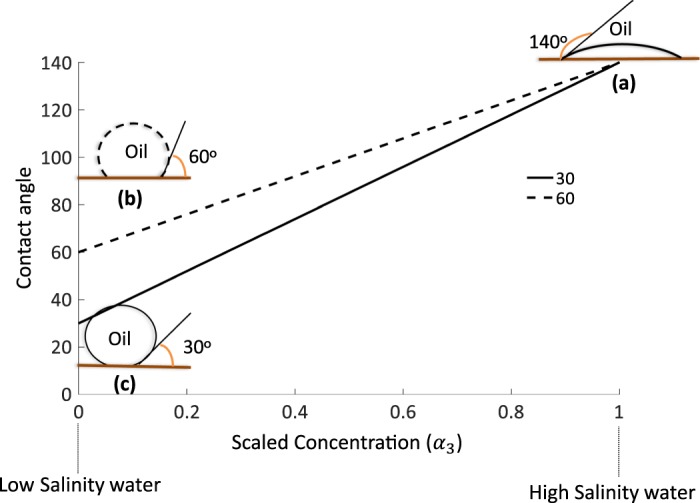
The linear equilibrium relation assumed between the contact angle (degree) and the scaled salinity (concentration). (**a**) Oil droplet on rock surface under equilibrium showing initial 140° contact angle. (**b,c**) Final wettability conditions of oil droplet on oil surface for both wettability alterations.

We assumed an equilibrium relation between concentration and contact angle as shown in Fig. 8. This relation does not take into account neither the chemical interactions at interfaces (e.g. interfacial tension change, visco-eleastic effects) nor the non-equilibrium effects (e.g. multi-ion exchange, double layer expansion)^10,20,26,63^, as the main purpose of this study is not to derive an expression between salinity and contact angle. The primary objective is to investigate the dynamics of two-phase flow induced by wettability alteration. We define two scenarios for the contact angle as a function of the scaled salinity in water as shown in Fig. 8. The scaled salinity has been defined by dividing the local concentration by the inlet concentration (${\alpha }_{3}(x,t)/{\alpha }_{3}(0,0)$). Values 1 and 0 represent the scaled high salinity and scaled low salinity conditions, respectively. At high salinity condition, contact angle is assumed to be 140° (oil-wet) and by decreasing the scaled salinity towards zero, the contact angle changes to water-wet condition. Two different scenarios for the water-wet case (contact angles of 60° and 30°) have been assumed based on a recent experimental study^20^.

### Hydrodynamic Transport of Low Salinity Water

Low salinity water will be transported into the porous media and possibly will mix with the high salinity water due to its miscible nature. To solve the transport of salinity within the water phase, the advection-diffusion equation has been solved, within the water phase (*α*_2_).

8 $$\frac{\partial {\alpha }_{3}}{\partial t}+\nabla \cdot ({\bf{u}}{\alpha }_{3}-{D}_{2,3}\nabla {\alpha }_{3})=0,{\rm{for}}\,{{\rm{\Omega }}}_{{\alpha }_{2}}$$

where *α*_3_ is the scaled salinity. The salinity is transported in water (*α*_2_) using advection and diffusion. The diffusion of salinity into oil (*α*_1_) has been set to zero to not allow the mass transfer of salinity from water to oil. To ease the numerical simulations, we inserted the term ${D}_{1,3}\nabla {\alpha }_{1}$, where ${D}_{1,3}$ was set to zero in the divergence term of Eq. 8 that resulted in a sharp interface between the scaled salinity (*α*_3_) and oil (*α*_1_). The diffusion coefficient of salinity in water was set to *D*_2,3_ = 10^−9^m^2^s^−1^ based on previous experimental study^7^. If Péclet number is defined based on the boundary values in our simulations the Péclet number can be up to a few hundreds. However, it is very small in the stagnant regions which is close to diffusion regime^44^.

We used the second-order schemes for both spatial and temporal terms. Variables were interpolated from cell centres to face centres using linear (central) interpolation and the van Leer limiter was applied to the convection terms to avoid numerical instabilities and improve accuracy.

### Numerical Domain, Boundary and Initial Conditions

**Figure 9:**
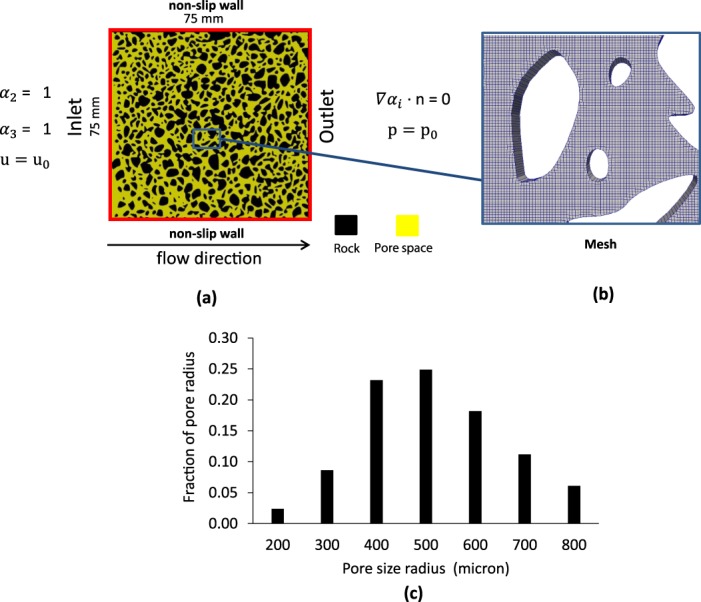
(**a**) 2D demonstration of the numerical domain, water (*α*_2_) and the scaled salinity (*α*_3_) were initially set to zero in internal domain, (**b**) a section of the mesh, (**c**) the medial axis pore-size distribution.

The numerical domain was a square two-dimensional geometry with the size of 75 mm × 75 mm and porosity of 0.58 and the average pore size of 500 micrometer, as shown in Fig. 9. Top and bottom boundaries are walls and share no-flow conditions with no-slip velocity. The right boundary is at constant pressure with zero gradient of scaled salinity (*α*_3_) normal to the boundary. The left boundary has constant inlet injection velocity and constant scaled salinity value. The injection rate is 0.05 ms^−1^. Water density (*ρ*_2_) and viscosity (*μ*_2_) were 998 kgm^−3^ and 10^−3^ and kgm^−1^s^−1^, respectively. Oil density (*ρ*_1_) and viscosity (*μ*_1_) were 844 kgm^−3^ and 1.910 × 10^−2^ kgm^−1^s^−1^, respectively. The interfacial tension (σ) was 0.07 kgs^−2^. Given these properties capillary number in our simulation was $Ca=7\times {10}^{-4}$, capillary-end effects can be ignored due to high capillary number. Capillary number is defined as $Ca=\frac{{\mu }_{w}{u}_{in}}{\sigma }$, in which *u*_*in*_ is the inlet velocity of water, *μ*_*w*_ is the viscosity of water and σ is the interfacial tension.

Three different initial conditions were used for low salinity waterflooding: i) the domain was fully saturated by the wetting phase (oil) and low salinity water injected, referred to the secondary mode, ii) high salinity water was injected at the given rate and the static contact angle between oil and high-salinity water was set 140°. After a steady-state saturation was reached, the low salinity water was injected at the same rate. This is referred to as the tertiary mode. iii) the same steady-state saturation obtained by high-salinity water flooding was chosen as the initial water saturation. However, the transport of low salinity water within the domain was not resolved and it was assumed that low salinity water will fill all the space filled by high-salinity water (no stagnant region). Then as a result the contact angle changed as a step function. This scenario, although is hypothetical, shows the dynamics of two-phase flow while the impact of stagnant regions were discarded.

The domain was meshed with OpenFOAM utility called snappyHexMesh^59^. The tool refines mesh around the grains to ensure a high-quality mesh in narrower pore throats. The mesh quality was ensured by doing a study of grid refinement, where number of cells in the narrowest pore were increased from 4 to 6, 8 and up to 25. The residuals, average velocity, and flux errors were calculated in each simulation to check the grid size convergence similar to the former study^44^. Simulations with 15 cells in narrowest pore throat, with same numerical schemes and solution, led to the smallest error (<1%) in mass balance calculations and further mesh size refinement did not decrease the error. As a result, the size of mesh for the whole pore space was 0.6 million cells.

### Generating Event Maps

To estimate the dynamics and pore-filling cycles during the whole simulations, event maps were generated. Event maps show the frequency of pore-filling events at every position within the pore space. For every two consecutive time steps, the value of *α*_1_ at each cell was checked. If at a given cell the value of *α*_1_ value changed from 1 to 0, it indicated a water-filling event and change of *α*_1_ from 0 to 1 indicated the oil-filling event. The changes were tracked from start of the simulation to the end of simulation, at the same time intervals. Then the number of times each cell went through oil and water filling events were summed up individually in each cell.

## Data Availability

After publication the data will be made available upon publication on the IMPRES group website (http://personalpages.manchester.ac.uk/staff/vahid.niasar/default.htm).
